# Acoustic Vowel Metrics as Correlates of Dysphagia and Dysarthria in Brainstem Neurodegenerative Diseases

**DOI:** 10.3390/audiolres15060152

**Published:** 2025-11-08

**Authors:** Silvia Capobianco, Luca Bastiani, Francesca Forli, Bruno Fattori, Francesco Stomeo, Maria Russo, Maria Rosaria Barillari, Andrea Nacci

**Affiliations:** 1 Otolaryngology, Audiology, and Phoniatrics Unit, University of Pisa, 56124 Pisa, Italy; 2 Institute of Clinical Physiology, National Research Council of Italy, Heart Hospital G. Pasquinucci, 54100 Massa, Italy; 3 Implant Section, Karolinska Institutet, 14186 Stockholm, Sweden; 4 Otolaryngology & Audiology Department, University Hospital of Ferrara, 44124 Ferrara, Italy; 5 Division of Phoniatrics and Audiology, Department of Mental and Physical Health and Preventive Medicine, University of L. Vanvitelli, 81100 Naples, Italy

**Keywords:** bulbar dysfunction, acoustic vowel analysis, neurogenic dysphagia

## Abstract

**Background/Objectives:** Swallowing and speech rely on shared brainstem circuits coordinating oropharyngeal motor functions. In neurodegenerative diseases affecting the brainstem—such as progressive supranuclear palsy (PSP), amyotrophic lateral sclerosis (ALS), and multiple system atrophy (MSA)—bulbar dysfunction often impairs tongue propulsion and motility, affecting both swallowing (dysphagia) and phonation (dysarthria). This study aimed to investigate whether vowel-based acoustic features are associated with swallowing severity in brainstem-related disorders and to explore their potential as surrogate markers of bulbar involvement. **Methods:** This was a cross-sectional observational study. Thirty-one patients (13 PSP, 12 ALS, 6 MSA) underwent clinical dysarthria assessment, acoustic analysis of the first (F1) and second (F2) formants during sustained phonation of /a/, /i/, /e/, and /u/, and swallowing evaluation using standardized clinical scales (DOSS, FOIS, ASHA-NOMS) and fiberoptic endoscopic evaluation (Pooling Score, Penetration-Aspiration Scale). The vowel space area (tVSA, qVSA) and Formant Centralization Ratio (FCR) were computed. **Results:** Significant correlations emerged between acoustic vowel metrics and dysphagia severity, especially for liquids. The FCR showed strong correlations with DOSS (ρ = −0.660, *p* < 0.0001), FOIS (ρ = −0.531, *p* = 0.002), ASHA-NOMS (ρ = −0.604, *p* < 0.0001), and instrumental scores for liquids: the Pooling Score (ρ = 0.538, *p* = 0.002) and PAS (ρ = 0.630, *p* < 0.0001). VSA measures were also associated significantly with liquid swallowing impairment. F2u correlated with dysarthria severity and all liquid-related dysphagia scores. **Conclusions:** Vowel-based acoustic parameters, particularly FCR and F2u, reflect the shared neuromotor substrate of articulation and swallowing. Acoustic analysis may support early detection and monitoring of bulbar dysfunction, especially where instrumental assessments are limited.

## 1. Introduction

The brainstem plays a fundamental role in the integration of processes involving the oro-pharyngo-laryngeal structures, acting as a neurophysiological integrator for the coordination of speech and swallowing, two tightly interwoven sensorimotor processes. Unlike purely cortical activities, both swallowing and speech rely on the precise orchestration of cranial nerve nuclei, descending motor tracts, ascending sensory afferents, and central pattern generators (CPGs) located within the medulla and pons [1,2].

Swallowing is governed by a distributed neural network, but its pharyngeal and esophageal phases are controlled largely involuntarily by medullary circuits. The swallowing central pattern generator (sCPG) is located in the dorsal medulla and is anchored by the nucleus tractus solitarius (NTS), which serves as the primary sensory hub for the swallowing reflex. The NTS receives afferent input from the oropharynx and larynx via cranial nerves IX (glossopharyngeal) and X (vagus) [2,3]. The NTS then projects to the nucleus ambiguus (NA), which drives motor output to the pharyngeal constrictors and laryngeal muscles [2]. This dorsal-ventral medullary loop, which links sensory reception to motor execution, is fundamental for triggering and executing the pharyngeal swallow reflex.

Moreover, the NTS integrates multiple sensory modalities (such as tactile, thermal, and chemical) from the oropharynx, larynx, and esophagus. These inputs modulate not only swallowing but also vocalization and breathing, indicating a shared sensorimotor platform between airway protection and phonation [4]. The reticular formation, through its medial and lateral columns, contributes to both the coordination and timing of these functions. On the motor side, the corticobulbar tract, descending from the lower precentral gyrus, synapses bilaterally on cranial nerve motor nuclei including V, VII, IX, X, XI, and XII, which innervate the muscles of articulation, phonation, and swallowing. The corticobulbar projections to cranial nerve nuclei are often asymmetrical, with the tongue and lower facial muscles receiving predominantly contralateral input. As a result, unilateral lesions of the brainstem or corticobulbar tract commonly lead to dysarthria and dysphagia due to impaired motor control on the opposite side [1,5].

Similarly to swallowing, voice production is shaped by the interplay of respiratory drive, glottic valving, and resonance control, each relying on distinct but interconnected brainstem pathways. The pre-Bötzinger complex, located in the ventrolateral medulla, generates the respiratory rhythm and synchronizes with laryngeal motoneurons in the NA to coordinate phonation with breathing [6]. The periaqueductal gray (PAG) in the midbrain further modulates vocal behavior based on emotional, volitional, or reflexive triggers, relaying to the reticular formation and pontomedullary nuclei [4].

**Figure 1:**
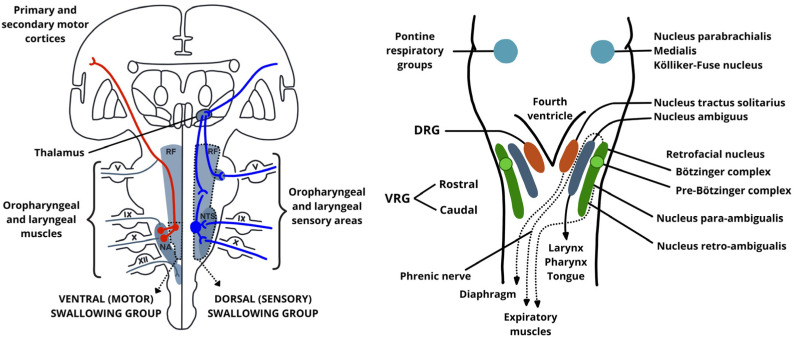
A schematic representation of the central control of swallowing and phonatory-respiratory coordination. (**Left panel**): The swallowing central pattern generator (sCPG) is organized into a dorsal sensory group (centered on the nucleus tractus solitarius, NTS) and a ventral motor group (centered on the nucleus ambiguus, NA). Afferent sensory input from the oropharynx and larynx (via cranial nerves IX and X) reaches the NTS, which integrates sensory information and projects to the NA to activate pharyngeal and laryngeal muscles. Cortical and subcortical motor inputs reach the brainstem via corticobulbar tracts. (**Right panel**): Respiratory and laryngeal motor output is coordinated via the ventral (VRG) and dorsal (DRG) respiratory groups. The pre-Bötzinger and Bötzinger complexes generate and modulate respiratory rhythm, interacting with nuclei involved in phonation and swallowing, including the NA and surrounding structures. This circuit ensures precise temporal integration between breathing, voice, and swallowing.

Due to the anatomical and functional proximity of the neural substrates responsible for voice and swallowing (Figure 1), damage to any of these structures, whether due to neurodegeneration, demyelination, or vascular insult, can disrupt the timing, strength, or coordination of motor output and result in dysphonia, dysarthria, and dysphagia, often simultaneously [2,7].

Acquired brainstem pathologies are frequently associated with impairments in swallowing and phonation due to the dense concentration of motor nuclei and interconnecting pathways within this region [1,4]. Damage to the corticobulbar tracts or cranial nerve nuclei—particularly those of the glossopharyngeal (IX), vagus (X), accessory (XI), and hypoglossal (XII) nerves—can result in the co-occurrence of dysphagia, dysarthria, and dysphonia. These symptoms may manifest acutely, as in brainstem stroke, or progressively, as seen in neurodegenerative diseases.

A paradigmatic example is the Lateral Medullary Syndrome (LMS or Wallenberg syndrome), a rare condition resulting from ischemia of the posterior inferior cerebellar artery (PICA), a branch of the vertebral artery, which leads to infarct of the lateral medulla. LMS manifests through a distinct constellation of neurological deficits. These symptoms include hemisensory disturbance (ipsilateral face, contralateral body), ipsilateral cerebellar signs, and ipsilateral Horner’s syndrome (ptosis, miosis, and anhidrosis). Additionally, the syndrome is characterized by dysphagia and dysarthria due to involvement of the nucleus ambiguus, with associated sensory and vestibulo-cerebellar symptoms reflecting broader medullary damage [8]. In very rare cases, dysphagia is described as the sole presenting symptom of the disease, leading to a diagnostic delay [9,10,11].

Brainstem stroke, particularly involving the medulla or lower pons, is one of the most common acquired causes of bulbar dysfunction. These lesions affect the oral and pharyngeal phases of swallowing, impairing bolus transport, airway protection, and laryngeal elevation. Dysarthria is often present and strongly correlates with oral-phase dysphagia severity, given the shared anatomical substrates of speech and swallowing [12,13,14].

In Amyotrophic Lateral Sclerosis (ALS), especially the bulbar-onset phenotype, early involvement of both upper and lower motor neurons leads to rapid deterioration of speech and swallowing functions. Objective measures such as reduced vowel space area (VSA), increased jitter and shimmer, and altered pitch dynamics are useful for identifying bulbar involvement even in the early stages of disease [14,15,16]. Recent studies using phonatory and time-frequency analysis have demonstrated high accuracy in detecting bulbar impairment in ALS patients using machine learning–based approaches [16].

In other conditions such as progressive supranuclear palsy (PSP), and multiple system atrophy (MSA), similarly to ALS, early bulbar signs often include both swallowing impairment and voice changes [16,17], reflecting the shared vulnerability of cranial motor pathways. Moreover, coordination among respiration, glottic closure, and pharyngeal movement, necessary for both safe swallowing and effective phonation, can be disrupted even by focal lesions within the brainstem [6,7].

Other clinical conditions, such as multiple sclerosis, brainstem tumors, autoimmune encephalitis, and traumatic brainstem injuries, may cause overlapping deficits depending on the structures involved. Given the functional interdependence of swallowing and vocal mechanisms, a comprehensive neurofunctional evaluation is essential for accurate diagnosis and targeted rehabilitation although early detection of bulbar symptoms remains challenging. Standard bedside swallowing assessments, while widely used, have limited sensitivity for subtle deficits and rely heavily on subjective interpretation [18]. Similarly, perceptual evaluation of dysarthria may overlook incipient articulatory or phonatory anomalies, especially in patients without overt speech complaints. Although instrumental assessments such as fiberendoscopic evaluation of swallowing (FEES), videofluoroscopy (VFS), and scintigraphy are essential for diagnostic confirmation, their availability is restricted by logistical and resource constraints [19,20].

In this context, voice analysis has emerged as a promising, non-invasive approach to support early identification of bulbar dysfunction. Recent work has highlighted potential correlations between acoustic voice features and swallowing dysfunctions, suggesting that vocal parameters may serve as surrogate markers of laryngeal competence and airway protection [21]. However, a lack of standardized protocols and robust normative data currently limits their implementation in clinical practice. Given the anatomical convergence, shared physiology, and overlapping symptoms, there is a growing rationale for developing integrated assessment tools that leverage vocal signals to infer bulbar integrity. This approach could prove especially useful in neurological populations where subclinical brainstem involvement precedes functional decline, and in settings where access to instrumental swallowing evaluations is limited.

The present study aims to explore acoustic voice features in a clinical population affected by specific subtypes of acquired brainstem diseases, investigating their potential association with swallowing impairment. By focusing on spectral properties of the voice signal—particularly vowel formants—we seek to characterize measurable vocal changes that may reflect underlying deficits in bulbar motor control. Our goal is to contribute to the development of accessible and objective tools for the early detection and monitoring of bulbar dysfunction.

## 2. Materials and Methods

### 2.1. Study Design and Setting

This was a cross-sectional observational study conducted in two tertiary referral hospital outpatient clinics: the Voice and Swallowing Disorders Outpatient Clinic, Pisa University Hospital (Pisa, Italy), and the Division of Phoniatrics and Audiology, Department of Mental and Physical Health and Preventive Medicine, University of Campania “L. Vanvitelli” (Naples, Italy). Both centers followed identical assessment protocols. The study was carried out between September 2024 and May 2025, and all assessments were performed in a single visit.

### 2.2. Inclusion and Exclusion Criteria

Eligible participants were consecutively recruited from patients referred to the outpatient swallowing clinics during the study period. Inclusion criteria were as follows: (1) diagnosis of progressive supranuclear palsy (PSP), amyotrophic lateral sclerosis (ALS), or multiple system atrophy (MSA) with bulbar involvement, confirmed by neurologists with specific expertise according to internationally accepted criteria; (2) age ≥ 18 years; (3) native Italian speaker; (4) the ability to undergo both acoustic voice analysis and fiberoptic endoscopic evaluation of swallowing (FEES); (5) the ability to provide informed consent. Exclusion criteria were as follows: (1) a history of head and neck cancer or surgery resulting in structural alterations of the oropharyngeal or laryngeal tract; (2) severe hearing loss; (3) previous speech-language therapy; (4) severe cognitive impairment preventing protocol completion; (5) incomplete data for any of the required acoustic or swallowing assessments.

The number of patients screened and excluded prior to enrollment was not systematically recorded; therefore, only participants meeting inclusion criteria and completing all assessments were included in the final analysis.

### 2.3. Variables

The exposure variables were acoustic parameters: first and second formant frequencies (F1, F2) for the vowels /a/, /i/, /e/, and /u/, total vowel space area (tVSA), quadrilateral vowel space area (qVSA), and Formant Centralization Ratio (FCR).

The primary outcome variables were clinical and instrumental measures of swallowing severity: Dysphagia Outcome and Severity Scale (DOSS), Functional Oral Intake Scale (FOIS), American Speech–Language–Hearing Association National Outcome Measurement System (ASHA-NOMS) swallowing scale, Pooling Score, and Penetration–Aspiration Scale (PAS).

The secondary outcome variable was dysarthria severity, assessed using the Radboud Dysarthria Assessment (RDA).

Potential confounders included age, sex, and neurological diagnosis. An overview of the study variables is provided in Table 1.

### 2.4. Data Sources and Measurement

#### 2.4.1. Acoustic Voice Analysis

Voice recordings were performed using the Kay Computer Speech Lab (CSL) 4500 system, connected to a PC and equipped with a Shure-Prolog SM48 microphone, positioned 15 cm from the subject’s mouth at a 45° angle. During the recording session, background noise was kept below 30 dB. The vocal samples, recorded digitally at a sampling rate of 50 kHz, were analyzed using version 2.3 of the MDVP 5105 software.

Before the recording, each patient underwent a short training session to ensure a stable phonation with minimal fluctuations in pitch and loudness. The training included three supervised practice trials with an experienced speech-language pathologist. Afterwards, patients were instructed to sustain the vowels /a/, /e/, /i/, and /u/ at a conversational intensity (55–65 dB) for at least 4 s.

The initial and final phases of each phonation were excluded from the analysis, and only the central portion of the vowel was considered. For each vowel, the first (F1) and second (F2) formants were extracted, resulting in the following parameters: F1a, F2a, F1e, F2e, F1i, F2i, F1u, and F2u.

##### Vowel Metrics

Vowel metrics are used to acoustically quantify articulatory performance and are derived from the first and second formant frequencies (F1 and F2). These formants represent spectral peaks shaped by the configuration of the vocal tract, particularly by the movement of the tongue body: F1 is inversely related to tongue height, while F2 increases with tongue frontness.

**Figure 2:**
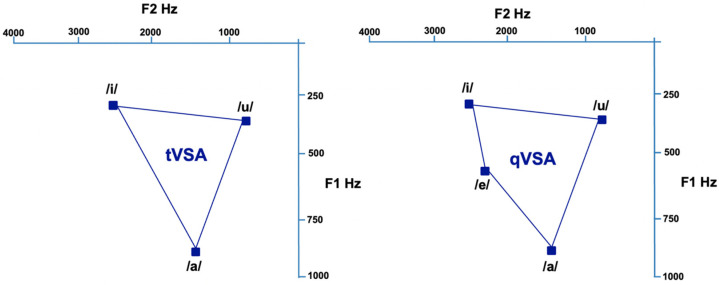
Visual representation of the triangular Vowel Space Area (tVSA, **left**) and quadrangular Vowel Space Area (qVSA, **right**) calculated in a cohort of 174 healthy subjects.

The triangular vowel space area (tVSA) and quadrangular vowel space area (qVSA) provide visual and quantitative estimates of the working range for vowel articulation. They are calculated from the Euclidean distances between the F1 and F2 coordinates of specific corner vowels plotted in the F1–F2 plane (Figure 2). The tVSA is based on the vowels /i/, /a/, and /u/, while the qVSA includes /i/, /e/, /a/, and /u/, according to the following formulas [22,23]:

(1) $$tVSA=0.5\times \left[F1i(F2a-F2u)+F1a\left(F2u-F2i\right)+F1u\left(F2i-F2a\right)\right]$$

(2) qVSA=0.5 ×∣(F1i×F2e+F1e×F2a+F1a×F2u+F1u×F2i)−(F2i×F1e+F2e×F1a+F2a×F1u+F2u×F1i)∣

To account for inter-individual variability [24] and to enhance the sensitivity to articulatory centralization [25], Sapir et al. (2010) proposed the Formant Centralization Ratio (FCR) [23]. This metric reflects the extent of formant collapse toward a central position in the vowel space, which is typically observed in dysarthric speech. It is calculated as follows:

(3) $$FCR=\frac{F2u+F2a+F1i+F1u}{F2i+F1a}$$

Following previous studies on vowel space metrics and the formulation of the Formant Centralization Ratio [23], we limited the analysis to corner vowels (/i/, /a/, /u/), which maximize the spread in the F1–F2 space and reduce inter-speaker variability. The mid-back vowel /o/ was excluded because of its partial overlap with /u/ in the F2 dimension and its greater articulatory variability, which may reduce metric stability.

#### 2.4.2. Clinical and Instrumental Evaluation of Dysphagia

All patients underwent a structured evaluation of oropharyngeal dysphagia, combining clinical scales and fiberoptic endoscopic assessment. The clinical evaluation included three standardized rating tools. The Dysphagia Outcome and Severity Scale (DOSS) is a 7-point scale that rates functional swallowing ability based on diet level, independence, and risk of aspiration, with lower scores indicating more severe impairment [26]. The Functional Oral Intake Scale (FOIS) assesses the patient’s oral intake on a 7-level continuum ranging from nothing by mouth to a full oral diet with no restrictions [27]. The ASHA-NOMS swallowing level scale, developed by the American Speech-Language-Hearing Association, reflects both diet consistency and the level of assistance required for safe feeding, also on a 7-point ordinal scale [28].

Instrumental evaluation was performed using Fiberoptic Endoscopic Evaluation of Swallowing (FEES), following standard protocols. Patients were administered food and liquid boluses of varying consistencies (thin liquids, semisolids, solids). Swallowing function was rated using two scales: the Pooling Score [3,19], which quantifies the amount and location of pharyngeal residue after swallowing, and the Penetration–Aspiration Scale (PAS), which measures the degree of airway invasion on an 8-point scale, from no penetration (score = 1) to silent aspiration (score = 8) [29].

These complementary tools provided a multidimensional profile of swallowing safety and efficiency across consistencies and tasks.

#### 2.4.3. Clinical Evaluation of Dysarthria

Dysarthria severity was assessed using the Radboud Dysarthria Assessment (RDA), a standardized tool validated for the evaluation of speech disorders in neurological populations [30]. The RDA comprises both speech tasks and observational ratings of speech subsystems, allowing for classification of dysarthria as absent, mild, moderate, or severe. All assessments were performed by experienced phoniatricians following the original validation protocol.

### 2.5. Study Size

The sample size was determined by convenience, including all eligible patients attending the two centers during the study period. No formal a priori sample size calculation was performed.

### 2.6. Statystical Analysis

All statistical analyses were performed using SPSS software (version 24.0; IBM Corp., Armonk, NY, USA) and STATA (version 15; StataCorp, College Station, TX, USA). A significance level of *p* < 0.05 was adopted for all inferential tests. Categorical variables were reported as percentages, and continuous variables were expressed as mean ± standard deviation (SD) or median with interquartile range (IQR), depending on data distribution. Descriptive statistics were first performed to summarize the clinical and demographic characteristics of the study population.

The chi-square test of independence was used to assess differences in the distribution of sex across dysarthria severity levels (mild, moderate, severe) and diagnostic groups. Age comparisons between males and females were conducted using independent-samples *t*-test. The Mann–Whitney U test was used to assess sex-related differences in acoustic vowel parameters (formant values, tVSA, qVSA, FCR), due to non-normal distributions.

Spearman’s rank correlation coefficients were computed to assess correlations between age, acoustic parameters, and swallowing scores and to explore the relationship between acoustic metrics and swallowing scores.

One-way analysis of variance (ANOVA) was applied to compare electroacoustic parameters (F1 and F2 for various vowels, tVSA, qVSA, FCR) and standardized dysphagia scores (Pooling for solid, semisolid, and liquid consistencies; PAS for the same consistencies; DOSS; FOIS; ASHA-NOMS) across diagnostic groups and dysarthria severity levels.

## 3. Results

### 3.1. Sample Characteristics

A total of 31 patients (18 females, 13 males; mean age = 68.03 ± 9.10 years; age range: 49–81 years) were included in the analysis. The cohort comprised 13 individuals with progressive supranuclear palsy (PSP), 12 with amyotrophic lateral sclerosis (ALS), and 6 with multiple system atrophy (MSA). Table 1 summarizes the demographic and clinical characteristics of the sample, including the distribution of dysarthria severity across the diagnostic groups.

### 3.2. Clinical Assessment of Dysarthria

All patients included in the study underwent the Radboud Dysarthria Assessment (RDA) [30], a standardized tool designed to evaluate the presence and severity of dysarthria based on both speech tasks and observational parameters.

According to the RDA, 2 patients showed no signs of dysarthria, while 11 were classified as having mild dysarthria, 8 as moderate, and 10 as severe. The distribution of dysarthria severity across diagnostic groups is reported in Table 2.

### 3.3. Effect of Age and Sex

To evaluate sex-related differences in acoustic parameters, a Mann–Whitney U test was applied. Statistically significant differences between males and females were observed for F1a (*p* = 0.043), F2a (*p* = 0.015), F2e (*p* = 0.015), and F2i (*p* = 0.025). No significant differences were found for the other formants or for the global vowel metrics tVSA, qVSA, and FCR (tVSA: *p* = 0.246; qVSA: *p* = 0.468; FCR: *p* = 0.769).

Chi-square tests were performed to assess potential associations between sex and dysarthria severity, diagnostic group, or age distribution. None of these comparisons showed statistically significant differences.

To assess sex-related differences in swallowing impairment, independent-samples *t*-tests were conducted for each of the dysphagia scores (DOSS, FOIS, ASHA-NOMS, Pooling, and PAS across consistencies). None of the comparisons reached statistical significance, indicating no effect of sex on swallowing impairment in the studied population.

The relationship between age and both acoustic and swallowing parameters was assessed using Spearman’s rank correlation coefficients. No significant correlations emerged between age and any of the acoustic vowel measures (tVSA: ρ = 0.145, *p* = 0.438; qVSA: ρ = 0.074, *p* = 0.692; FCR: ρ = −0.099, *p* = 0.595). Among dysphagia scores, age was only significantly correlated with PAS scores for solids (ρ = −0.368, *p* = 0.042). No other significant associations were observed.

### 3.4. Acoustic and Swallowing Measures Across Dysarthria Severity Levels

An ANOVA with Bonferroni-corrected post hoc comparisons was conducted to investigate differences in acoustic vowel metrics across dysarthria severity levels. For this analysis, patients with absent and mild dysarthria were pooled together, due to the low number of subjects in the “absent” category (n = 2).

**Figure 3:**
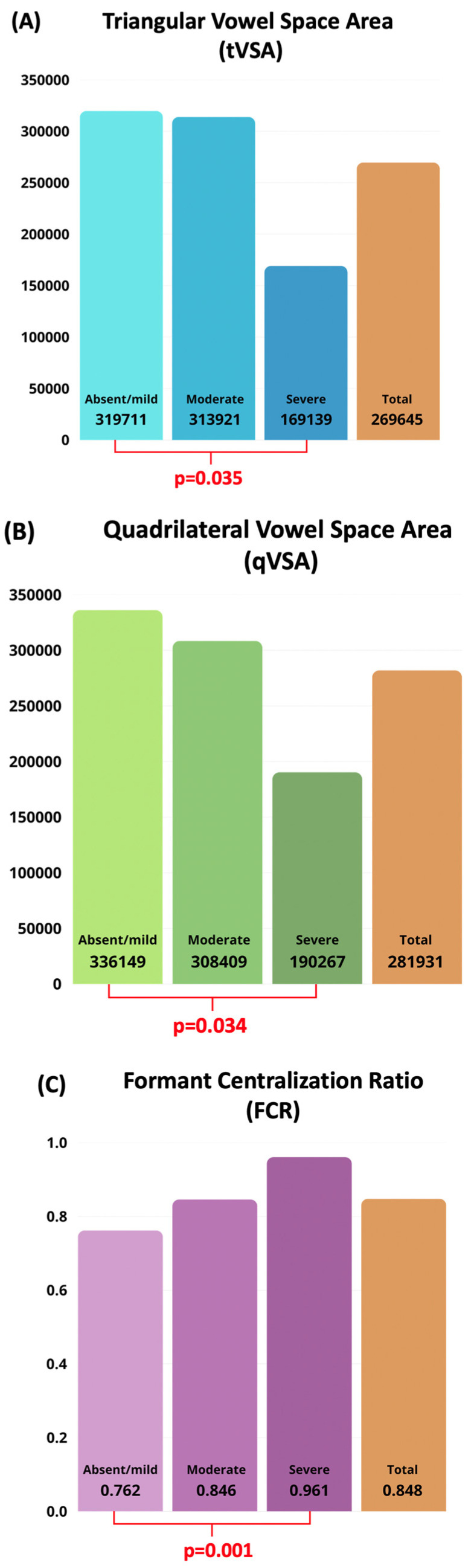
(**A**–**C**) The distribution of acoustic vowel metrics across dysarthria severity levels (Absent/Mild, Moderate, Severe) and in the overall sample (Total). Patients with absent and mild dysarthria were grouped together due to the low number of asymptomatic cases. (**A**) The triangular Vowel Space Area (tVSA, Hz^2^); (**B**) the quadrilateral Vowel Space Area (qVSA, Hz^2^); (**C**) the Formant Centralization Ratio (FCR, unitless).

The analysis revealed statistically significant differences in all three global vowel measures, tVSA (*p* = 0.035), qVSA (*p* = 0.034), and FCR (*p* = 0.001), with a progressive reduction in vowel space areas and an increase in FCR from absent/mild to severe dysarthria (see Figure 3A–C).

Among individual formants, F2u was the only parameter showing a statistically significant difference between absent/mild and severe dysarthria (*p* = 0.006).

One-way ANOVA with Bonferroni-corrected post hoc tests was performed to explore differences in swallowing scores according to dysarthria severity. A statistically significant effect was found for the Pooling score with liquid consistencies (ANOVA *p* = 0.013). Post hoc comparisons revealed significantly higher scores in the severe dysarthria group compared to both the mild group (*p* = 0.032) and the moderate group (*p* = 0.036). No significant differences were observed for the other dysphagia scales across dysarthria severity levels.

### 3.5. Variance Analysis by Diagnosis

A one-way ANOVA with Bonferroni-corrected post hoc tests was conducted to assess differences in acoustic parameters and swallowing scores among the three diagnostic groups (ALS, PSP, and MSA). A statistically significant effect of diagnosis was observed for the Pooling score with solid consistencies (ANOVA *p* = 0.029). Post hoc analysis revealed that patients with ALS showed significantly higher Pooling scores compared to both those with PSP (*p* = 0.039) and MSA (*p* = 0.045).

Regarding acoustic features, significant differences across diagnoses were observed in specific formant frequencies. F1a was significantly lower in the MSA group compared to ALS (*p* = 0.035), while F2a was significantly higher in PSP compared to MSA (*p* = 0.046). Additionally, F1i differed significantly between ALS and PSP (*p* = 0.036). On the contrary, no significant differences were found among the diagnostic groups for global vowel space measures (tVSA, qVSA, FCR) or for other swallowing scores.

### 3.6. Correlation and Association Analyses

Spearman’s rank correlation coefficients were computed to assess the relationship between acoustic vowel metrics and swallowing severity scores. Among global acoustic parameters, FCR showed significant correlations with multiple dysphagia scales, including DOSS (ρ = −0.660, *p* < 0.0001), FOIS (ρ = −0.531, *p* = 0.002), ASHA-NOMS (ρ = −0.604, *p* < 0.0001), Pooling score for liquids (ρ = 0.538, *p* = 0.002), and PAS for liquids (ρ = 0.630, *p* < 0.0001). Vowel space area parameters also significantly correlated with dysphagia scores: tVSA showed a significant correlation with DOSS (ρ = 0.407, *p* = 0.023), Pooling score for liquids (ρ = −0.360, *p* = 0.047) and PAS score for liquids (ρ = −0.556, *p* = 0.001). Similarly, qVSA correlated with DOSS (ρ = 0.408, *p* = 0.023), Pooling score for liquids (ρ = −0.414, *p* = 0.021) and PAS score for liquids (ρ = −0.554, *p* = 0.001).

Among individual formants, F2u correlated significantly with DOSS (ρ = −0.557, *p* = 0.001), FOIS (ρ = −0.531, *p* = 0.002), ASHA-NOMS (ρ = −0.551, *p* < 0.001), Pooling score for liquids (ρ = 0.529, *p* = 0.002), and PAS for liquids (ρ = 0.541, *p* = 0.003) (Table 3).

Moreover, significant correlations were found among the dysphagia scores themselves and among the acoustic parameters, supporting the internal coherence of each assessment domain.

## 4. Discussion

The main objective of the study was to investigate possible correlations between acoustic articulation parameters, derived from the frequency of the first (F1) and second formant (F2) for various vowels, and the presence of dysphagia, assessed through both clinical and instrumental methods, in order to identify a potential common biomarker of oral motor dysfunction. Particular attention was given to the role of the tongue in a population affected by neurodegenerative diseases of the brainstem and characterized by different degrees of articulatory and swallowing impairments.

In the descriptive analyses, a significant difference in formant frequencies was observed based on sex. This finding was expected, as previous studies have shown that formant frequencies are influenced by gender [31,32]. Men, having a longer and wider vocal tract, typically exhibit lower formant frequencies compared to women and children, whose shorter vocal tracts result in higher formant frequencies [33]. This difference in formant frequency directly impacts the vowel space area (VSA). However, no sex-related differences were found for the global metrics of articulation (tVSA, qVSA, FCR). In particular, the Formant Centralization Ratio (FCR) was proposed by Sapir and colleagues in 2010 specifically to minimize the influence of sex when studying formant centralization [23].

Age did not show a significant effect on the analyzed acoustic parameters, in contrast to what has been reported in previous studies on neurodegenerative diseases [32]. This may be due to the relatively young mean age of the study population (68.03 ± 9.10 years) and to the fact that the underlying neurological diseases had a greater impact on articulatory and swallowing function than age-related degeneration.

Among dysphagia scores, age was significantly correlated only with PAS scores for solids, which is consistent with clinical descriptions of presbyphagia, typically characterized by increased pharyngeal residue for solid consistencies and a higher risk of penetration and aspiration [34].

The clinical severity of dysarthria, assessed using the Radboud Dysarthria Assessment (RDA) [30], was significantly correlated with formant-based acoustic markers of articulatory impairment (tVSA, qVSA, and FCR). Among these, FCR showed the strongest association, confirming its particular sensitivity to articulatory deficits, in line with previous findings [23,35].

In our study, acoustic indices of dysarthria, particularly FCR and vowel space area (tVSA, qVSA), showed significant correlations with both clinical and instrumental measures of dysphagia severity, reinforcing the growing body of evidence supporting a close relationship between dysarthria and oropharyngeal dysphagia in neurodegenerative brainstem disorders. Both processes share critical bulbar motor pathways, particularly those governing tongue, laryngeal, and pharyngeal movements, and the co-occurrence of these symptoms is well documented across various neurological conditions, including stroke, Parkinson’s disease, ALS, and other neuromuscular diseases [12,13,36,37]. However, the underlying neurophysiological overlap and the possibility of predicting one symptom from the other remain underexplored. The present findings are consistent with previous studies showing that reduced articulatory working space and formant convergence—considered markers of dysarthria—are also associated with oropharyngeal swallowing deficits [14,15,38]. The observed correlations, particularly with oral-phase impairment and penetration/aspiration risk, support the hypothesis that tongue base dysfunction contributes to both articulatory breakdown and impaired bolus propulsion.

In our study, both clinical severity of dysarthria and acoustic vowel metrics showed a significant association with dysphagia for liquids, as measured by both the Pooling Score and PAS scale. This finding highlights a specific vulnerability of liquid bolus control in patients with severe bulbar motor impairment. Liquids, due to their low viscosity and rapid flow, require highly coordinated movements of the oral and pharyngeal structures to ensure safe transit and airway protection [39,40]. Even minimal delays or inaccuracies in lingual propulsion, velopharyngeal closure, or laryngeal elevation can result in pre- or intra-swallowing penetration or aspiration [39,41]. As observed in patients with progressive supranuclear palsy, impaired tongue base retraction and delayed initiation of the pharyngeal swallow contribute disproportionately to penetration and aspiration of thin liquids [39]. Similarly, in amyotrophic lateral sclerosis, early tongue dysfunction has been shown to impact both articulation and bolus propulsion, with liquids being more likely to elicit silent aspiration due to their lower sensory feedback and faster transit [39,42]. In this context, dysarthria, particularly when severe, may reflect a global impairment of bulbar motor coordination rather than merely reduced strength [21]. As such, acoustic signs of articulatory disruption may serve as early proxies of impaired coordination affecting not only speech but also the handling of liquids during swallowing [16,21].

Among all formant parameters, F2 of the vowel /u/ was the only one to show consistent and significant correlations with both clinical and instrumental measures of dysphagia, as well as with clinical dysarthria severity.

This finding may be explained by the particular articulatory configuration required to produce /u/, which involves an elevated and posterior tongue position, combined with lip protrusion. The second formant (F2) reflects the anteroposterior placement of the tongue, with lower values characterizing vowels produced with a more posterior tongue position, such as /u/, compared to more anterior vowels like /i/. Importantly, this tongue configuration closely resembles the posture required for the propulsive phase of swallowing, where an effective posterior lingual thrust is necessary to initiate bolus transit from the oral cavity to the pharynx. A dysfunction in this mechanism, often present in bulbar syndromes, could therefore affect both speech articulation, as measured by acoustic analysis of F2u, and swallowing efficiency. This interpretation is consistent with the hypothesis formulated by Rusz and colleagues [43], who proposed that the second formant of /u/, by reflecting posterior tongue positioning, may be particularly sensitive to early articulatory impairment and valuable for characterizing motor deficits in dysarthric speech.

Acoustic analysis of voice offers a non-invasive, objective tool to support early detection of bulbar dysfunction. Compared to clinical or instrumental swallowing assessments, it is faster, repeatable, and less resource-intensive, making it suitable for longitudinal monitoring or screening in settings with limited access to specialized care. Previous studies have highlighted that acoustic features may serve as surrogate markers of laryngeal and pharyngeal coordination, thus complementing traditional assessments and potentially guiding timely intervention strategies [16,21]. In the future, integration of acoustic measures into digital platforms or smartphone-based applications could enable remote screening and personalized follow-up in neurological populations.

This study presents several limitations that should be acknowledged. First, the sample size was relatively small and unbalanced across diagnostic groups, which may limit the statistical power and generalizability of the findings. Additionally, the lack of a healthy control group or a disease control cohort without dysphagia makes it difficult to assess the specificity of acoustic markers for bulbar dysfunction. Moreover, the cross-sectional design, combined with the absence of longitudinal follow-up, precludes any conclusions about the temporal dynamics of acoustic changes or their potential role in tracking disease progression or treatment response. Another potential limitation is the heterogeneity of the underlying neurological diagnoses, which may differ in their pattern and severity of bulbar involvement. The potential influence of disease-related fatigue during the assessments could also have affected both speech and swallowing performance. Furthermore, the possible impact of medications (particularly those acting on the central nervous system) was not specifically controlled for and could have influenced motor function in some participants. Finally, the use of isolated vowel production, while methodologically consistent, may not fully capture the complexity of phonatory behavior in natural speech contexts.

## 5. Conclusions

This study supports the potential utility of acoustic vowel analysis as a complementary tool for assessing bulbar motor function in patients with brainstem-related neurodegenerative diseases. By focusing specifically on conditions characterized by prominent brainstem involvement, such as ALS, PSP, and MSA, we targeted a clinical population where early detection of bulbar dysfunction is both critical and challenging. The observed correlations between vowel-based spectral parameters and clinical measures of dysphagia severity highlight a shared neuromotor vulnerability between speech and swallowing systems. Given its non-invasive nature and ease of administration, acoustic analysis may serve as an accessible adjunct to traditional clinical assessments, particularly in settings where instrumental evaluations are not readily available. However, given the observational design, limited sample size, and other study limitations, these findings should be interpreted with caution. Further large-scale, prospective, and high-quality clinical trials are warranted to confirm these associations, establish their predictive value, and refine the role of acoustic analysis in early screening and monitoring of bulbar involvement across neurological populations.

## Figures and Tables

**Table 1:** An overview of study variables, including their role in the analysis and the corresponding measurement tools, following the STROBE (Strengthening the Reporting of Observational Studies in Epidemiology) checklist. Abbreviations: VSA = Vowel Space Area; FCR = Formant Centralization Ratio; DOSS = Dysphagia Outcome and Severity Scale; FOIS = Functional Oral Intake Scale; ASHA-NOMS = American Speech-Language-Hearing Association—National Outcome Measurement System; PAS = Penetration-Aspiration Scale; RDA = Radboud Dysarthria Assessment; PSP = Progressive Supranuclear Palsy; ALS = Amyotrophic Lateral Sclerosis; MSA = Multiple System Atrophy.

Category	Variable(s)	Role in Analysis	Measurement Tool/Method
**Exposure**	Formant frequencies (F1, F2) for /a/, /i/, /e/, /u/; total VSA (tVSA), quadrilateral VSA (qVSA), Formant Centralization Ratio (FCR)	Main exposure variables	Acoustic voice analysis from sustained vowels
**Primary outcome**	Dysphagia severity (DOSS, FOIS, ASHA-NOMS); instrumental swallowing measures (Pooling Score, PAS)	Primary dependent variables	Clinical scales; Fiberoptic Endoscopic Evaluation of Swallowing (FEES)
**Secondary outcome**	Dysarthria severity	Secondary dependent variable	Radboud Dysarthria Assessment (RDA)
**Confounders**	Age, sex, diagnosis (PSP, ALS, MSA)	Potential confounding factors	Recorded from clinical records

**Table 2:** Demographic data and dysarthria severity by diagnosis (mean age ± standard deviation; dysarthria severity distribution; total number of patients, N). PSP: Progressive Supranuclear Palsy; ALS: Amyotrophic Lateral Sclerosis; MSA: Multiple System Atrophy.

Diagnosis	Mean Age	Absent-Mild Dysarthria	Moderate Dysarthria	Severe Dysarthria	N
PSP	73.31 ± 6.03	4	4	5	13
ALS	64.25 ± 10.79	6	3	3	12
MSA	64.17 ± 5.38	3	1	2	6
**TOTAL**	**68.03 ± 9.10**	**13**	**8**	**10**	**31**

**Table 3:** Spearman’s rank correlation coefficients (ρ) and p-values for the association between acoustic parameters and swallowing outcomes. tVSA: triangular vowel space area; qVSA: quadrilateral vowel space area; FCR: formant centralization ratio; F2u: second formant frequency of vowel /u/. ρ: Spearman’s correlation coefficient; *p*: significance value.

Acoustic Parameter	DOSS (ρ, *p*)	FOIS (ρ, *p*)	ASHA-NOMS (ρ, *p*)	Pooling for Liquids (ρ, *p*)	PAS for Liquids (ρ, *p*)
tVSA	ρ = 0.407 *p* = 0.023	ρ = 0.216 *p* = 0.242	ρ = 0.329 *p* = 0.071	ρ = −0.360 *p* = 0.047	ρ = −0.556 *p* = 0.001
qVSA	ρ = 0.408 *p* = 0.023	ρ = 0.244 *p* = 0.186	ρ = 0.327 *p* = 0.073	ρ = −0.414 *p* = 0.021	ρ = −0.554 *p* = 0.001
FCR	ρ = −0.660 *p* = <0.0001	ρ = −0.531 *p* = 0.002	ρ = −0.604 *p* = <0.0001	ρ = 0.538 *p* = 0.002	ρ = 0.630 *p* = <0.0001
F2u	ρ = −0.557 *p* = 0.001	ρ = −0.531 *p* = 0.002	ρ = −0.551 *p* = <0.001	ρ = 0.529 *p* = 0.002	ρ = 0.541 *p* = 0.003

## Data Availability

The data presented in this study are available on request from the corresponding author due to privacy restrictions.
